# 2023 Management Recommendations of Bangladesh Rheumatology Society on Pharmacological Treatment of Rheumatoid Arthritis With Synthetic and Biologic Disease-Modifying Drugs

**DOI:** 10.7759/cureus.59395

**Published:** 2024-04-30

**Authors:** Muhammad Shoaib Momen Majumder, A.T.M. Tanveer Hasan, Minhaj Rahim Choudhury, Shamim Ahmed, Md. Titu Miah, Md. Robed Amin, Md. A Shahin, Ariful Islam, Md. Nahiduzzamane Shazzad, M. Masudul Hassan, Abul Khair Ahmedullah, Md. Mujibur Rahman, Sabrina Yesmeen, Taslim Uddin, Syed A Haq

**Affiliations:** 1 Rheumatology, Bangabandhu Sheikh Mujib Medical University, Dhaka, BGD; 2 Rheumatology, Green Life Medical College and Hospital, Dhaka, BGD; 3 Internal Medicine, Directorate General of Medical Education, Mohakhali, Dhaka, BGD; 4 Internal Medicine, Directorate General of Health Services (DGHS), Dhaka, BGD; 5 Internal Medicine, Popular Medical College, Dhaka, BGD; 6 Rheumatology, BIRDEM (Bangladesh Institute of Research and Rehabilitation in Diabetes, Endocrine and Metabolic Disorders) General Hospital, Dhaka, BGD; 7 Rehabilitation Medicine, Bangabandhu Sheikh Mujib Medical University, Dhaka, BGD

**Keywords:** pharmacologic treatment, disease-modifying drugs, management recommendation, bangladesh rheumatology society, rheumatoid arthritis

## Abstract

Rheumatoid arthritis (RA) is the most common inflammatory polyarthritis in Bangladesh. Bangladesh Rheumatology Society (BRS) proposes these management recommendations to treat the considerable burden of RA in the resource-constrained situation based on the best current evidence combined with societal challenges and opportunities. BRS formed a task force (TF) comprising four rheumatologists. The TF searched for all available literature, including updated American College of Rheumatology (ACR), European Alliance of Associations for Rheumatology (EULAR), and Asia‐Pacific League of Associations for Rheumatology (APLAR) and several other guidelines, and systematic literature reviews until October 2023, and then a steering committee was formed, which included rheumatologists and internists. We followed the EULAR standard operating procedures to categorize levels of evidence and grading of recommendations.

This recommendation has two parts -- general (diagnosis of RA, nomenclature of disease-modifying anti-rheumatic drugs [DMARDs], disease activity indices) and management portion. The TF agreed on four overarching principles and 12 recommendations. Overarching principles deal with early diagnosis and disease activity monitoring. Recommendations 1-5 discuss using glucocorticoids, NSAIDs, and conventional synthetic DMARDs (csDMARD). Recommendations 6-9 stretch the use of targeted synthetic DMARDs (tsDMARDs) and biological DMARDs (bDMARDs). The suggested DMARD therapy includes initiation with methotrexate (MTX) or another csDMARD (in case of contraindication to MTX) in the first phase and the addition of a tsDMARD in the second phase, switching to an alternative tsDMARDs or bDMARDs in the subsequent phases. The TF included the Padua prediction score for the thromboembolism risk estimation. Recommendations 10-12 cover infection screening, vaccination, and DMARD tapering. Bangladesh has a higher prevalence of RA. This recommendation will serve as a tool to treat this high burden of patients with RA scientifically and more effectively.

## Introduction and background

Rheumatoid arthritis (RA) is a common inflammatory polyarthritis that mainly involves small peripheral joints in a symmetrical pattern that may cause deformities, leading to significant disability. The global prevalence of RA is 0.5-1%, and there is an increasing trend in females [1]. Bangladesh has a higher prevalence (1.6%) of RA, whereas the estimates of RA in the neighboring countries are 0.5% in India, 0.6% in Pakistan, and 0.1% in Thailand [2]. RA causes a substantial economic burden as well as significant health impacts. Bangladesh is the eighth most populous country globally, home to more than 174 million people [3]. So, in a resource-constrained country like Bangladesh, the impact of RA on health and the economy may be colossal. Therefore, patients’ and physicians’ awareness and availability of most of the disease-modifying anti-rheumatic drugs (DMARDs), including newer ones, have recently improved the outcomes of the disease.

To date, multiple DMARDs with different mechanisms of action have been used. The application of DMARDs in RA is to halt or slow the natural course of erosion and articular damage, preventing deformity and disability. Smolen JS proposed a new nomenclature for DMARDs in 2013, which broadly classifies DMARDs into two types: synthetic and biologic DMARDs [4]. Synthetic DMARDs are again divided into conventional synthetic DMARDs (csDMARDs) and targeted synthetic DMARDs (tsDMARDs).

The leading rheumatology bodies, like the Asia‐Pacific League of Associations for Rheumatology (APLAR), the European Alliance of Associations for Rheumatology (EULAR), the American College of Rheumatology (ACR), and several other groups and countries, formulated their guidelines for RA management. The Bangladesh Rheumatology Society (BRS) reviewed all the updated references. This management recommendation aims to treat the patients suffering from RA in a resource-constrained country like Bangladesh scientifically and effectively on the background of high infection rates, including tuberculosis (TB).

## Review

Methods

The Executive Committee (EC) of BRS formed a task force (TF) that comprised four rheumatologists. The TF searched titles, abstracts, and full text of the retrieved articles utilizing several search engines, including MEDLINE (by PubMed), Web of Science, Scopus, Directory of Open Access Journals, and Google Scholar, from January 2010 to October 2023, and relevant articles published in English. The TF formulated overarching principles (OP) and recommendations based on evidence from the relevant articles. Several rounds of discussion occurred among the members of TF. They categorized levels of evidence and strength of recommendations according to the most updated EULAR standard operating procedures for evaluating recommendations (supplementary description; supplementary tables 7, 8) [5]. The EC also formed a steering committee (SC) consisting of 12 members, which included rheumatologists and internists, experts in treating rheumatic diseases.

The OP and recommendations were finalized through a voting panel utilizing the modified Delphi approach to achieve the consensus. The members of the TF and SC participated in the voting, provided feedback and opinions, and suggested some modifications. The differences were discussed and resolved. Subsequently, a poll was launched to provide their levels of agreement (LoA) with the proposed recommendation, allowing them to choose any from the five options: 1 - accept/agree, 2 - accept/agree with some reservations, 3 - accept/agree with major reservations, 4 - reject with some reservations, and 5 - reject completely. A draft statement was endorsed as the final recommendation when the combined percentages of the responses for "accept/agree" and "accept/agree with some reservations" achieved ≥75% of votes among the attendees. The accepted recommendations (including respective strengths) were prepared in a tabulated form and sent for voting on the LoA using a numeric rating scale of 0-10 (0 indicating no agreement at all, 8-9: strongly agree, and 10 indicating full agreement). The TF and SC again substantiated consensus on ≥80% agreement. Then, two more email rounds were arranged to reach the final consensus. The mean LoA, the standard deviation (SD), and the percentage of votes for eight or more for each item are provided in the recommendation table. 

Recommendations

General Part

The RA diagnosis is mainly clinical, presenting with symmetrical inflammatory polyarthritis (morning stiffness for at least 30 minutes) predominantly involving the small joints aided by physical examination and relevant investigations. Before reaching the final diagnosis of RA, the important differentials should be excluded at least by history and clinical examination, e.g., psoriatic arthritis (self or family history of psoriasis), peripheral spondyloarthritis (history of inflammatory back pain, enthesitis, self or family history of painful red eye), enteropathic arthritis (abdominal pain, weight loss, or rectal bleeding), systemic lupus erythematosus (photosensitivity, oral ulcer, history of fetal loss), or Sjogren syndrome (dryness of mouth or grittiness of eyes). The ACR/EULAR classification criteria were proposed in 2010 (supplementary description; Supplementary table 9) [6].

The knowledge and management of RA have improved dramatically in the last several years. Treat to target (T2T) is the philosophy of treatment. The primary approach is to achieve either low disease activity (LDA) or remission by judicious use of DMARD in the early part of the disease. The nomenclature of different DMARDs and disease activity indices are mentioned in Tables 1, 2, respectively.

**Table 1:** DMARD nomenclature DMARD, disease-modifying anti-rheumatic drug.

DMARD nomenclature	Synthetic DMARDs	Conventional synthetic (csDMARDs)	Methotrexate, leflunomide, sulfasalazine, hydroxychloroquine
		Targeted synthetic (tsDMARDs)	Tofacitinib, baricinitib, upadacinitib, peficinitib, filgotinib
	Biological DMARD	Biological originator DMARDs	TNF inhibitors: etanercept, adalimumab, golimumab, infliximab, certolizumab pegol; B cell (CD20) inhibitor: rituximab; IL-6 inhibitor: tocilizumab, sarilumab; co-stimulation blocker: abatacept
		Biosimilar DMARDs	Adalimumab, etanercept, rituximab

**Table 2:** Rheumatoid arthritis disease activity indices DAS28: disease activity score based on 28 joints; ESR: erythrocyte sedimentation rate; CDAI: clinical disease activity index.

Thresholds of disease activity	Instruments to measure [7]	
	DAS28 (ESR)	CDAI
Remission	<2.6	≤2.8
Low disease activity (LDA)	≥2.6 to ≤3.2	>2.8 to ≤10
Moderate disease activity (MDA)	>3.2 to ≤5.1	>10 to ≤22
High disease activity (HDA)	>5.1	>22

Several prognostic factors are used to make treatment decisions and assess the prognosis of disease severity. The EULAR first incorporated poor prognostic factors into major therapeutic decision changes in 2010. BRS suggests any poor prognostic factors as the decision criteria mentioned below.

Poor Prognostic Factors

Moderate (after csDMARD like MTX therapy for at least three months) to high disease activity according to composite measures {CDAI>10 [8], DAS 28>3.2 [9]}, high acute phase reactant levels, e.g., erythrocyte sedimentation rate (ESR)≥52 mm/h [10], C-reactive protein (CRP)≥10 mg/L [9], high joint activity (≥4 tender joints, ≥4 swollen joints) [9], and presence of RF and or anti-CCP Ab, especially at high titers (≥3 ULN [upper limit of normal]) [11].

The aim of the treatment of RA is not only pain reduction but also the prevention of joint damage and functional disability. Various composite tools have been validated to measure disease activity in RA patients, such as DAS28, CDAI, simplified disease activity index, and ACR response criteria (ACR20, ACR50, and ACR70). BRS recommends CDAI and DAS28 to monitor RA disease activity for the Bangladeshi population. Both DAS28 and CDAI are based on 28 joint counts. CDAI has some advantages over DAS28, like being simple and easier and not requiring any laboratory investigations like ESR or CRP. The categorization of disease activity states based on validated tools is mentioned in Table 2.

Overarching Principles

The purpose of treatment is to provide the greatest care for people with RA. The disease course, prognosis, therapeutic options, and outcome should be explained to the patients. The treatment target is to achieve either remission or LDA, considering the poor prognostic factors and treatment costs, which is problematic for a resource-constrained country like Bangladesh. RA treatment needs a holistic approach requiring drug treatment, disease monitoring, management of comorbidities, psychological, social and occupational support, rehabilitation for deforming arthritis, and, in selected cases, orthopedic surgery. The BRS management recommendations are mentioned in Table 3. 

**Table 3:** BRS rheumatoid arthritis treatment recommendations cs/tsDMARD, conventional synthetic/targeted synthetic disease-modifying anti-rheumatic drugs; bDMARD, biological disease-modifying drug; CoE, category of evidence; SoR, strength of recommendation; LoA, level of agreement. The hash signs (#, ##) have been used to separate portions of individual recommendation statements with different evidence categories and, consequently, different corresponding recommendation grades. ^§^LoA: Refers to the mean (±SD) of the levels of agreement provided by the voting panel members. ^¶^%LoA ≥8: Refers to the percentage of the voting panel members who expressed a level of agreement ≥8.

Overarching principles		CoE	SoR	§LoA	¶%LoA ≥8
1	The diagnosis of RA should be made as early as possible, and a DMARD should be initiated early to prevent irreversible joint damage.	3	C	9.6±0.7	100
2	The pharmacological treatment of RA should be done by rheumatologists^#^ and internists^## ^(preferably experienced in treating rheumatic diseases).	3^#^, 4^##^	C^#^, D^##^	9.2±1.3	94.7
3	The aim of RA treatment is to achieve a good quality of life and maintain physical functioning by achieving remission or low disease activity in every patient.	1A	A	9±1.2	94
4	All patients having active disease should be monitored frequently (every 2 to 6 weeks), and therapy should be adjusted if there is no improvement by 3 months after initiation of treatment or the target is not achieved by 6 months.	2B	B	8.8±1.5	94.7
Recommendations					
1	Methotrexate should be the anchor drug for RA unless contraindicated.	1A	A	9.7±0.7	100
2	Sulfasalazine or leflunomide can be used as a first-line treatment strategy for RA patients who have a contraindication or early intolerance to MTX.	1A	A	9.4±0.9	100
3	NSAIDs should be used at therapeutic doses for initial therapy of patients with active RA unless contraindicated.	4	D	8.9±1.4	94
4	Glucocorticoid monotherapy is not recommended.	1A	A	9.8±0.5	100
5	Short-term low-dose GC (≤7.5 mg) can be used while initiating treatment in patients with moderate to severe disease while waiting for the effects of csDMARD, changing csDMARD or experiencing flares.	1A	A	9.3±0.9	100
6	The addition of tsDMARD (JAK inhibitor) should be considered in the presence of poor prognostic factors if treatment with MTX (or other csDMARDs) fails to achieve the treatment goal. Switching to other csDMARD (leflunomide or sulfasalazine) can be considered without poor prognostic factors.	1A^#^, 4^##^	A^#^, D^##^	8.6±1.1	83.33
7	Padua Prediction Score can be used to estimate the risk of thromboembolism while using or escalating the dose of tsDMARD. BRS suggests infection screening before using tsDMARD.	4	D	8.3±1.4	82
8	The options of a second tsDMARD (JAK inhibitor) versus bDMARD should be explained to patients who fail to a first tsDMARD to reach a shared decision.	4	D	8.7±1.3	93.75
9	All the targeted therapies, including tsDMARDs and bDMARDs, should be combined with MTX or other csDMARDs except in case of contraindication or intolerance to MTX or other csDMARDs.	1A	A	9.3±0.8	100
10	Screening for active or latent tuberculosis infection (LTBI) and chronic hepatitis B and C virus infection should be considered before initiating tsDMARD^#^ or bDMARD^##^. BRS recommends combining chest X-ray and QuantiFERON-TB gold assay to screen for latent tuberculosis.	1A^#^, 2A^##^	A^#^, B^##^	9.2±0.9	94
11	Vaccination is recommended in all RA patients before starting DMARDs or biologics.	Uncategorized	Ungraded	9.5±0.8%	100
12	The treatment of RA should be continued for an indefinite period. Tapering of DMARD should only be considered if the RA target (remission or low disease activity) is maintained for at least six months.	1B	A	9.7±0.5	100

OP 1: Early Diagnosis of RA

The diagnosis of RA should be made as early as possible, and a DMARD should be initiated early to prevent irreversible joint damage. A Swiss clinical trial on early treatment initiation (<1 year of symptom onset) versus late treatment (1-5 years of symptom onset) revealed that early treatment initiation resulted in a long-lasting reduction in radiological progression beyond over five years [12]. If RA patients are left untreated, significant radiological damage (more than 80% having joint space narrowing on plain radiography) can occur within the first two years of symptom onset [13]. Physical disability is higher in those whose treatment has been delayed.

OP 2: Holistic Approach of RA Management

Management of RA should be done by rheumatologists and internists (preferably experienced in treating rheumatic diseases). Treatment of RA needs a holistic approach, and the management is complex. This is important because rheumatologists and dedicated internists are already aware that starting treatment early can help prevent joint damage, lower the need for orthopedic surgery (if treatment starts within three months of the first sign of symptoms), and address different complications (interstitial lung disease, neuropathy, etc.) and comorbidities [14]. Rheumatologists have vast experience in treatment using csDMARD and bDMARD regarding dosing and managing adverse effects [15]. We have included internists trained in rheumatology as there is a paucity of rheumatologists in many districts and hospitals of Bangladesh.

OP 3: T2T Strategy

The aim of RA treatment is to achieve a good quality of life and maintain physical functioning by achieving remission or LDA in every patient. Many diseases like diabetes, hypertension, and dyslipidemia have therapeutic targets that reduce organ failure and mortality. Functional decline in RA significantly increases death [16]. The "T2T" strategy has improved RA patients’ outcomes, and different clinical trials use the T2T strategy with newer molecules. The T2T expert group suggested remission (DAS28 <2.6 or CDAI <2.8) or LDA (DAS28 2.6 and 3.2 or CDAI >2.8 and 10) to prevent joint deterioration, physical impairment, premature death, and improve quality of life [17]. Remission is for DMARD-naïve patients and LDA for those who failed earlier treatments, have a long-standing disease, are elderly, or have multiple comorbidities [15]. Remission reduces cardiovascular (CV) risk and osteoporosis [18,19]. Achievement of remission or LDA may often necessitate bDMARDs or tsDMARDs, which may be unaffordable for some patients in Bangladesh. Many patients are engaged in physical labor, which may worsen RA symptoms or make mechanical components predominate; concomitant fibromyalgia may make achieving an LDA state difficult. These patients need disease education, counseling, and drug compliance to manage symptoms and function.

OP 4: Regular Monitoring

All patients with active disease should be monitored frequently (every two to six weeks), and therapy should be adjusted if there is no improvement by three months after the initiation of treatment or if the target is not achieved by six months. For relative improvement by three months and remission or LDA by six months, patients with high/moderate disease activity should be monitored monthly [20]. A 50% improvement in a disease activity assessment instrument (e.g., CDAI/DAS28 from baseline to three months) indicates a better disease activity or relative progress. Minor improvement after three months limits attaining the aim at six months, making it a crucial milestone to measure therapy response. A meaningful improvement by this stage suggests remission or LDA by six months [21], and disease activity at three and six months strongly correlates with that at the end of one year [22]. BRS suggests a 50% decrease in DAS28 or CDAI at three months to monitor improvement and goal attainment in the next three months. Treatment can be modified by optimizing MTX (or other csDMARD) dose or route (e.g., switching from oral to SC route) or intra-articular glucocorticoid (GC) injections in the presence of one or few residual active joints [15]. Despite some benefits of the optimum MTX dose, moderate to high disease activity after three months requires a change in therapy [23]. If the patient is near the target at the end of six months, the treatment may be continued for a few more weeks, as some patients may achieve the target shortly after six months. Poor economic status is a significant obstacle to frequent RA disease activity monitoring. A model letter addressing the local physicians can be used regarding dose escalation of MTX and follow-up investigations.

Management recommendations

Recommendation 1

Methotrexate (MTX) should be the anchor drug for RA unless contraindicated.

Based on the safety, efficacy, easy dosing schedule, and low cost, MTX remains the cornerstone of treatment for most RA patients, either as monotherapy or in combination. MTX and GC combination showed an efficacy almost similar to MTX combined with different bDMARDs in treating early RA [24,25]. MTX combination with other csDMARDs was not superior to MTX monotherapy for early RA treatment [26]. Janus kinase (JAK) inhibitors for csDMARD-naïve patients are not FDA-approved. MTX reduces CV mortality by 70% and reduces joint replacement surgery, suggesting chondroprotection [27,28]. For a 70-year-old patient with moderately active disease, the initial dose can be 7.5 mg once weekly. For a relatively younger patient with highly active disease and weighing >50 kg, MTX 15 mg once weekly can be initiated, while for those weighing <50 kg, MTX 10 mg can be initiated [29]. The MTX dose should be rapidly escalated by 5 mg at two- to four-week intervals. The target dose is 25 mg/weekly and is well tolerated in Bangladeshi people [30]. In case of intolerance to more than 15 mg/week or unsuccessful at this dose, subcutaneous (SC) MTX or splitting the oral dose every 12 hours on the same day each week should be tried. SC MTX is more effective than oral MTX at the same dose with fewer gastrointestinal side effects [31]. Before commencing bDMARDs, SC MTX may be cost-effective if oral MTX is ineffective or intolerable [32]. To decrease MTX toxicities and enhance compliance, folic acid (1-5 mg) should be supplemented daily, excluding the MTX day [29]. Weekly 5 mg folinic acid (alternative to folic acid) can be administered 12 hours after MTX. Coffee or dark chocolate with MTX also reduces adverse events. One cup of coffee (1.5 teaspoons) synchronized with the MTX weekly dose in the morning, repeated 1-3 hours before MTX at dinner and the next morning. Coffee dislikers can substitute four 40-gram squares of at least 50% dark chocolate 1 hour before MTX. If intolerance persists, another 40 grams of chocolate can be taken 8-12 hours after MTX [33,34]. Caffeine inhibits CNS adenosine receptors, improving MTX tolerance [34]. MTX should be stopped at least one month before pregnancy. Stop MTX immediately and start 5 mg of folic acid daily in unplanned pregnancies [35]. After three months of optimized MTX treatment, patients with continued high disease activity should discontinue MTX and switch to another DMARD.

Recommendation 2

Sulfasalazine (SSZ) or leflunomide (LEF) can be used as a first-line treatment strategy for RA patients who cannot tolerate or have a contraindication to MTX.

A systematic review comparing the efficacy of different csDMARDs showed that MTX (15 mg/week) was better than SSZ and had equal efficacy to LEF [36]. MTX had less disease progression after two years than LEF [37]. MTX is contraindicated in pregnancy or contemplating pregnancy, lactation, liver disease (transaminases >3 ULN, serum bilirubin >5 mg/dL), excessive alcohol intake, and renal impairment (CrCl <30 mL/min) [29]. LEF is preferred to SSZ for treating moderate to severe RA because of its potentially greater efficacy [38]. LEF is a 20 mg single daily dose, cheaper than SSZ. LEF can be used in patients of all stages of renal dysfunction [39]. SSZ (up to 3 g/day) can be used in patients for whom LEF is contraindicated, such as patients with liver disease. Both SSZ and hydroxychloroquine (HCQ) are considered safe in pregnancy and lactation [35]. SSZ can be administered at the standard dose of up to the estimated glomerular filtration rate (eGFR) of 30 mL/min and reduced by 50% below this level, usually 500 mg in hemodialysis patients (maximum 1000 mg) [40]. In patients with RA, HCQ has a beneficial effect on reducing CV events by decreasing modifiable factors such as lipid profile, glycemic status, and diabetes incidence. HCQ can be added to those at cardiovascular disease (CVD) risk [41]. HCQ is well tolerated and has minimal risk of toxicity, suitable for patients with mild disease (e.g., seronegative RA without erosions) who cannot afford routine follow-up.

Recommendation 3

NSAIDs should be used at full therapeutic doses for initial therapy of patients with active RA unless contraindicated.

NSAIDs are used temporarily to control disease symptoms in patients in whom treatment with DMARDs is being started. A full dose of NSAID should be continued for at least two weeks before switching to another NSAID. Effective anti-inflammatory doses include 1000 mg of naproxen, 150 mg of indomethacin (cheaper), and 120 mg of etoricoxib. Naproxen, ibuprofen (provided not on aspirin), or celecoxib is preferred for the elderly and cardiac patients [42]. Non-selective cyclooxygenase (COX) inhibitors can be continued till the 30th week of pregnancy and lactation [43]. A selective COX-2 inhibitor (e.g., etoricoxib) and aceclofenac may be suitable for people with NSAID-induced gastrointestinal side effects or concomitant GC use. Adjuvant analgesics can be used in very active diseases and renal impairment. Patients who need frequent NSAIDs or analgesics despite adequate inflammation control should be searched for associated fibromyalgia.

Recommendation 4

Oral GC monotherapy is not recommended.

None of the countries or recommending bodies approved GC as the sole agent. The most common adversely affected systems by GC in RA patients are psychological and behavioral disturbances (e.g., minor mood disturbances), dermatological system (cutaneous atrophy, acne, alopecia, ecchymosis), and CV system (dyslipidemia, atherosclerosis, hypertension, edema, CVD) followed by infections [44]. In Bangladesh, self-medication of GC is rampant due to a lack of proper education, low cost, and lack of monitoring by drug authorities, posing a severe threat to steroid-induced adverse effects. Misuse of corticosteroids by some physicians is not uncommon. BRS recommends developing awareness among clinicians on using GC and assessing a suspected RA case by a rheumatologist or an internist dedicated to treating rheumatic diseases before initiating GC.

Recommendation 5

Short-term low-dose GC (≤7.5 mg) can be used while initiating treatment in patients with moderate to severe disease waiting for the effects of csDMARD, changing csDMARD or experiencing flares.

GC should be tapered as soon as clinically feasible. GC rapidly reduces inflammation in active RA, unlike csDMARDs. BRS prefers low-dose GC due to some current clinical research and to avoid GC abuse in Bangladesh. Low-dose prednisolone is defined as ≤7.5 mg/day in Europe and ≤10 mg/day in the US [7,15]. The recent GLORIA (Glucocorticoid Low-dose in Rheumatoid Arthritis) provided strong evidence for adding low-dose prednisolone (5 mg/day) to control disease activity and delay joint damage progression [45]. Another study found that low-dose short-term GC (≤10 mg/day) improved clinical severity in most early RA patients [46]. GC therapy can be used as bridging therapy while changing csDMARD. Short-term GC use is preferable for ≤3 months and not exceeding six months [15]. DMARD therapy may be considered a failure if GC cannot be withdrawn within six months [15]. Except for severe extra-articular symptoms such as vasculitis, neuropathy, diffuse parenchymal lung disease, or scleritis, BRS opposes oral, IV, and IM moderate- to high-dose GC. These patients should be referred to a rheumatologist.

We recommend prednisolone ≤7.5 mg/day (up to 10 mg can be considered), then 5 mg/day, and finally 2.5 mg/day, using each dose for 2-4 weeks. Rarely, patients with disabling arthritis may be treated with the tREACH trial GC protocol (weeks 1-4: 15 mg/day, weeks 5-6: 10 mg/day, weeks 7-8: 5 mg/day, and weeks 9-10: 2.5 mg/day) [47]. Intraarticular injections of GCs (e.g., triamcinolone acetonide) can decrease synovitis in residual active or reactivated joints. The procedure requires aseptic precautions. Resting large weight-bearing joints for 24-48 hours after injection maximizes benefits. Blood glucose, lipid profile, and bone mineral density should be monitored in patients on GC for more than six months. Exercise, calcium, vitamin D, and bisphosphonates (in proper cases) can reduce the incidence of GC-induced osteoporosis.

Recommendation 6

The addition of tsDMARD (JAK inhibitor) should be considered in the presence of poor prognostic factors if treatment with MTX (or other csDMARDs) fails to achieve the treatment goal.

**Figure 1:**
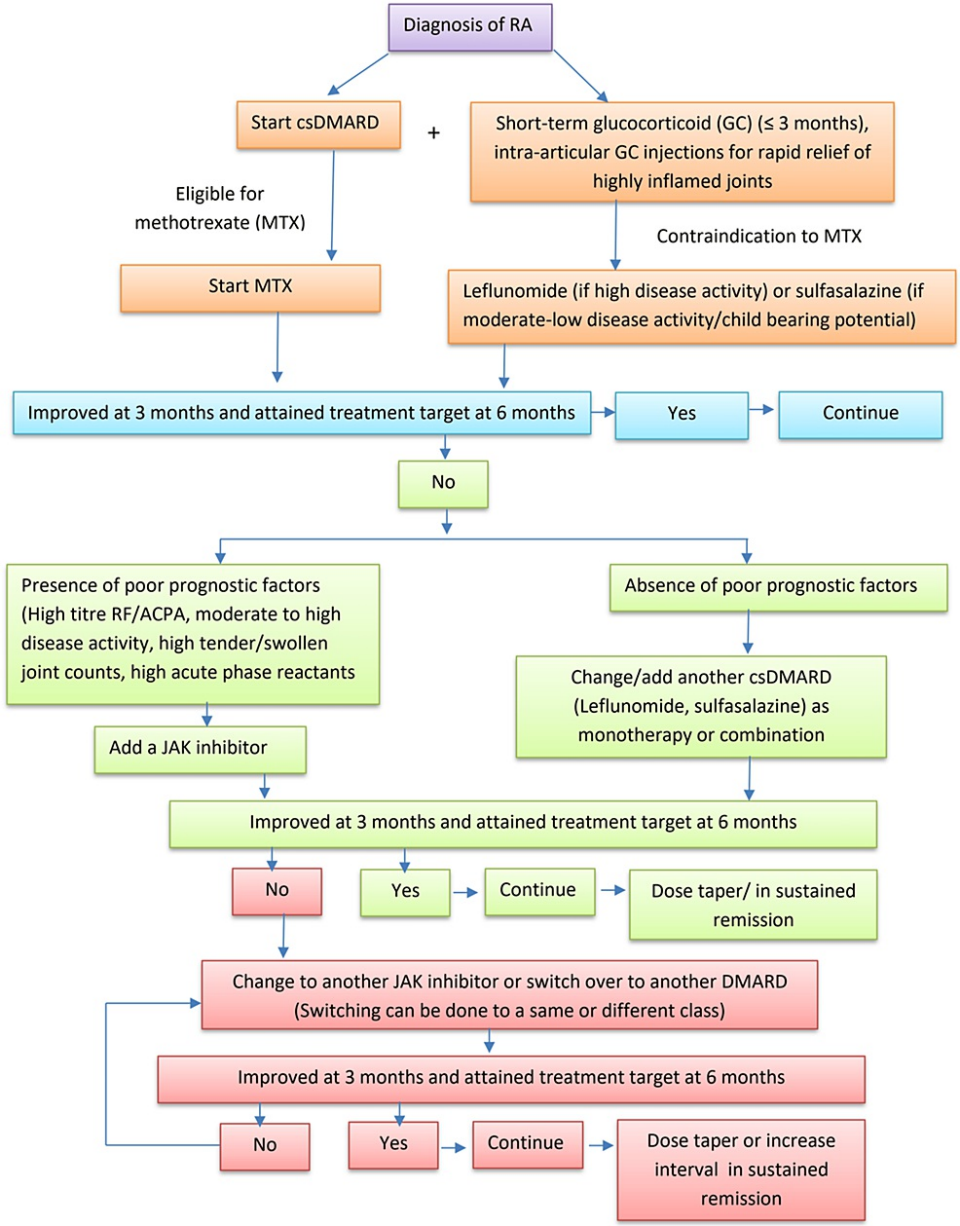
Flowchart for RA management Use 2010 ACR/EULAR RA criteria to diagnose RA. The different color schemes in the algorithm represent consecutive stages of RA management. The color brown represents the first stage of managing rheumatoid arthritis (RA), while the color green denotes the second stage when the initial treatment with MTX (or other csDMARD) fails. The color pink is used to depict the third stage of therapy for patients who do not respond well to the second stage. csDMARD, conventional synthetic DMARD; JAK inhibitor, Janus kinase inhibitor; bDMARD, biological disease-modifying drugs. Image credit: Muhammad Shaoib Momen Majumder.

Switching to other csDMARD (LEF or SSZ) can be considered in patients without poor prognostic factors. In the presence of poor prognostic factors, the EULAR 2019 RA update recommended using tsDMARD or bDMARD [20]. Current guidelines recommend either bDMARD or tsDMARD. Poor prognostic indicators indicate severe disease progression, radiological damage, and physical disability [9]. Early bDMARD treatment in patients with poor prognostic factors improves clinical outcomes, prevents joint damage, and improves the quality of life [9]. The tsDMARDs inhibit radiological progression similarly [48]. Since generic tsDMARDs are cheaper and oral, we recommend them before bDMARDs (Figure 1). Despite the excellent initial response, secondary treatment inefficacy can occur with anti-TNF and other biologics (due to anti-drug antibodies) [49]. The secondary loss of treatment efficacy has not yet been observed with the tsDMARDs [49]. The EULAR 2019 TF concluded that tsDMARD and bDMARD are equally effective [20].

Tofacitinib is 5 mg twice a day. Tofacitinib can be used up to 15 mg daily because 20 mg increases the risk of thromboembolic events [50]. Tofacitinib should not be started if hemoglobin (Hb) is <8 g/dL or a significant infection occurs [51]. Tofacitinib was safe over 9.5 years in the most extensive clinical dataset for RA JAK inhibitors. This largest safety analysis showed similar safety event rates to other JAK inhibitors and bDMARDs except for herpes zoster (HZ) reactivation [52]. Baricinitib showed clinically significant improvement compared to placebo and adalimumab (RA-BEAM study) [53]. Baricitinib was more efficacious as a first-line tsDMARD in biologic DMARD-naïve patients in a Japanese multicenter registry [54]. This supports the BRS recommendation to use JAK inhibitors before bDMARDs. The SELECT-BEYOND research found that upadacitinib was beneficial and showed rapid improvement in bDMARD refractory RA patients (three or more bDMARDs) [55].

In the absence of poor prognostic factors, consider switching to another csDMARD. Most Bangladeshis have poor prognostic factors (high RF, anti-CCP antibody, and acute phase reactants). The BRS TF prefers changing to a different csDMARD (LEF or SSZ) as there is a lack of data about effective combination therapy except for triple therapy (MTZ+SSZ+HCQ) [56]. There is no difference in efficacy between LEF monotherapy and LEF combination therapy (MTX+LEF, LEF+HCQ, LEF+MTX+HCQ) [1]. HCQ with MTX may increase MTX bioavailability and anti-inflammatory effects [56]. MTX and SSZ combination can be considered in patients with a risk of infection or other comorbidities.

Recommendation 7

Padua Prediction Score can be used to estimate the risk of thromboembolism while using or escalating the dose of tsDMARD. BRS suggests infection screening before using tsDMARD. JAK inhibitors may increase the risk of venous thromboembolism (VTE)-including deep venous thrombosis (DVT), pulmonary embolism (PE), and arterial thromboembolism (ATE). Tofacitinib 10 mg twice a day increases these risks in those with baseline CV and VTE risk factors. Age ≥50, current smoker, HDL <40 mg/dL, hypertension, diabetes, and history of coronary artery disease diagnosis are baseline CV risk factors [57]. VTE risk factors include age ≥60, smoking, prior heart failure, history of VTE (DVT or PE), obesity (BMI >30 kg/m^2^), and oral contraceptives [57]. Several risk factors are responsible for these adverse events, such as immobility, thrombophilic conditions (e.g., antiphospholipid syndrome, malignancy, etc.,), and prednisolone ≥7.5 mg/day [58]. Upadacitinib had a similar safety profile compared to adalimumab for major adverse cardiovascular events (MACE), VTE, and malignancies. There is no dose relationship between upadacitinib (15 vs 30 mg) and VTE development. Patients should be educated to report acute shortness of breath, pleuritic chest pain, unilateral limb edema, and erythema (PE or DVT). Before switching to 15 mg tofacitinib or 4 mg baricinitib, BRS suggests checking for parameters mentioned earlier or Padua Prediction Score (Table 4). The Padua Prediction Score assesses the risk of VTE in hospitalized patients and identifies high-risk patients who may need thromboprophylaxis. As the tsDMARDs are cheaper in Bangladesh, there is a higher propensity to use these molecules; we need a cheaper thromboembolic prediction score for those at risk. The Padua Prediction Score (Table 4) is cheap (no investigations required) and includes most risk factors; thus, the BRS TF considers it. Padua score <4 suggests low VTE [59] risk and allows using a larger JAK inhibitor dose (15 mg tofacitinib or 4 mg baricinitib) when the standard dose fails.

**Table 4:** Padua Prediction Score Source: [59]. VTE, venous thromboembolism.

Items	Score
Active cancer (local or distant metastasis and/or chemotherapy in the previous 6 months)	3
Previous VTE (with the exclusion of superficial vein thrombosis)	3
Reduced mobility	3
Thrombophilia	3
Recent (≤1 month) trauma and/or surgery	2
Elderly years (≥70 years)	1
Heart and/or respiratory failure	1
Acute myocardial infarction or ischemic stroke	1
Acute infection and/or rheumatologic disorder	1
Obesity (≥30 kg/m^2^)	1
Ongoing hormone treatment	1

According to a meta-analysis, JAK inhibitors are unlikely to enhance the incidence of VTE compared to placebo [60]. Most VTE incidents occurred in the long-term extensions of clinical trials with prolonged exposure to treatment with CV or VTE risk factors [57]. RA patients with high disease activity have an enhanced risk of VTE than those in remission. JAK inhibitors are used for active RA that has not responded to previous treatments, making it hard to determine their true impact [61]. Huss et al. found no increased cancer risk in 69,308 RA patients, and 658,589 person-years treated with biologics or tsDMARDs (largest observational study) [62]. The Safety of TofAcitinib in Routine Care Patients with Rheumatoid Arthritis (STAR-RA) multi-database, population-based study of 102,263 RA patients (larger than ORAL surveillance trial and CORRONA RA registry study) found no increased risk of CV outcomes with tofacitinib in the real world [63]. The ARTIS trial (Anti-Rheumatic Therapies in Sweden), a 10-year large cohort, found no difference in MACE incidence between tsDMARDs and bDMARDs [64]. Data from the German RABBIT register shows that tsDMARDs did not show a high frequency of MACE compared to other DMARDs [65]. No increased incidences of VTE or MACE were observed in long-term, open-label extension studies of baricitinib and upadacitinib [66]. We did not observe any MACE or VTE in our study population treated with tofacitinib [67].

The JAK inhibitors are associated with a heightened risk of infection, particularly HZ reactivation. Baricinitib 2 mg and upadacitinib 15 mg seem to carry a lower risk than tofacitinib 5 and 10 mg (extrapolated from different phase II and III clinical trials) [68]. Concomitant GC use, tofacitinib 10 mg twice daily [69], age ≥65 years, and baricitinib 4 mg daily [68] are associated with a higher risk of HZ infection. HZ infections are usually nonserious and treatable with standard antivirals [69]. JAK inhibitors should be stopped during the infection period. Vaccination reduces varicella-zoster virus risk. Table 5 provides detailed information on the dosage of various Jak inhibitors and their application in specific circumstances.

**Table 5:** Dosing of different Jaknibs in normal and special circumstances Source: [58]. CrCl: creatinine clearance; JAKi: Janus kinase inhibitor.

Drug	Usual dose	Elderly population	Hepatic impairment	Renal impairment
Tofacitinib (first-generation JAKi) [70]	5 mg twice daily, highest 15 mg/day	Avoid >65 years of age	Mild impairment (Child-Pugh A): No adjustment; Moderate impairment (Child-Pugh B): 5 mg OD; Severe impairment (Child-Pugh C): Not recommended	CrCl <30 mL/min: 5 mg once daily ESRD requiring hemodialysis: Administer after dialysis session on dialysis days, 5 mg OD [51]
Baricitinib (first generation) [58]	2 to 4 mg once daily	Patients over 75 years: 2 mg	Contraindicated on severe impairment (Child-Pugh C)	CrCl 30-60 mL/min: 2 mg; CrCl <30 mg/mL: Contraindicated
Upadacinitib (second-generation JAKi) [70]	15 mg	Patients over 65 years: 15 mg	Mild to moderate impairment (Child-Pugh class A or B): No adjustment necessary. Contraindicated on severe impairment (Child-Pugh C)	No dose adjustment is currently recommended [70]

BRS insists on taking a customized history of fever and focal infections and conducting a clinical examination to find focal signs of infections before initiating JAK inhibitors. Monitor complete blood count, transaminases, and renal function at months 1 and 3, then every three months. A lipid profile should be done once at three months [58]. Dyslipidemia detected during this period should be treated according to international guidelines (e.g., American Heart Association [71], European Society of Cardiology [72], and Lipid Association of India [73]). RA patients have a high CV risk, compared to diabetes. Statins should be used to reduce CV risk, assessed by online calculators such as ASCVD Risk Estimator Plus [74] or QRISK3-2018 risk calculator [75].

Recommendation 8

The options of a second tsDMARD (JAK inhibitor) versus bDMARD should be explained to patients who fail to respond to a first tsDMARD to reach a shared decision. The EULAR 2019 RA management recommendation suggested trying baricitinib if tofacitinib didn’t work or vice versa [20]. Changing from one JAK inhibitor to another JAK inhibitor is called JAK inhibitor cycling [14]. Our country’s perspective demands JAK inhibitor cycling since biologics are expensive. A second JAK inhibitor may succeed if the first fails due to differences in JAKi selectivity, drug-specific bioavailability, and tissue penetrance [14]. Those who fail multiple tsDMARDs or refuse a second trial should switch to a bDMARD. Non-anti-TNF bDMARDs (rituximab) may be preferable in Bangladesh owing to lesser reactivation of latent tubercular foci and less costly than anti-TNF. Several non-inferiority trials showed that retreatment with a single dose of 1000 mg rituximab (at least 24 weeks interval) was effective and was associated with a lower incidence of infection [76]. Tocilizumab (IL-6 receptor antibody) may be preferred to rituximab for those at risk of serious infection [77].

MTX and calcineurin inhibitor (cyclosporine [CSA] or tacrolimus) combination has been explored in RA [78,79]. CSA can also be used with SSZ or LEF [80]. Another benefit of CSA is that it can be used in patients with hepatic diseases [81]. In patients who have failed tofacitinib or baricitinib with MTX and cannot afford biologics, BSR weakly suggests a six-month study with MTX (LEF alternative) plus CSA or tacrolimus. Treatment with the triple therapy regimen (MTX plus SSZ plus HCQ) can also be used in patients for the above scenario, which may be non-inferior to biologics [82].

Recommendation 9

All the targeted therapies, including both tsDMARDs and bDMARDs, should be combined with MTX or other csDMARD except in case of contraindication or intolerance to MTX. Adding MTX to targeted therapy (tsDMARD or bDMARD) provides a favorable clinical and radiological outcome compared to the targeted therapy alone [83]. MTX (LEF if intolerant) inhibits the development of anti-drug antibodies that impair bDMARD efficacy. The ORAL strategy trial showed that the tofacitinib and MTX combination was non-inferior to the adalimumab and MTX combination. In contrast, tofacitinib monotherapy failed to demonstrate non-inferiority to the adalimumab and MTX combination [70]. Baricitinib-MTX combination decreased joint damage more than MTX monotherapy [84]. Upadacitinib-MTX combination had higher remission than the tofacitinib-MTX combination [85].

Recommendation 10

Screening for active or latent tuberculosis infection (LTBI) and chronic hepatitis B and C virus infection should be considered before initiating tsDMARD or bDMARD. Bangladesh is a high TB burden country. The total prevalence of all forms of TB in Bangladesh was 429,800 in 2017, and the overall national TB prevalence for all forms of TB was 260 per 100,000 in Bangladesh in 2016 [86]. The conservation of granuloma (composed of immune cells and *Mycobacterium tuberculosis*) is essential to contain bacteria. TNF signaling aids mycobacterial defenses. Anti-TNF treatment disrupts TNF-mediated immunity, reactivating TB [87]. JAK inhibitors may inhibit TNFγ production by T cells by blocking IL-12 or IL-23, which act through JAK2/TYK2 [88]. Prophylactic treatment for latent TB reduces active TB incidences in patients using bDMARDS or JAK inhibitors.

Since LTBI has no symptoms, screening is essential. Compared to healthy controls, 40% of Indian RA patients were tuberculin skin test (TST)-positive [89]. We assume a similar picture due to proximity and demographics. BRS recommends chest X-ray (CXR), TST (using 10 PPD), and the interferon-gamma release assay (IGRA) blood test (e.g., QuantiFERON-TB Gold plus assay) to screen for LTBI. Quantiferon TB Gold has 98% specificity in the BCG-vaccinated population and is not affected by the patient’s BCG vaccination status [87]. Due to its high cost, IGRA may only be a suitable LTBI screening test for some in Bangladesh. BRS recommends CXR and TST with a cutoff of ≤5 mm due to the evidence of a greater agreement between TST and IGRA when the 5 mm cutoff is adopted, reducing the chance of false-negative TST [90]. 

**Figure 2:**
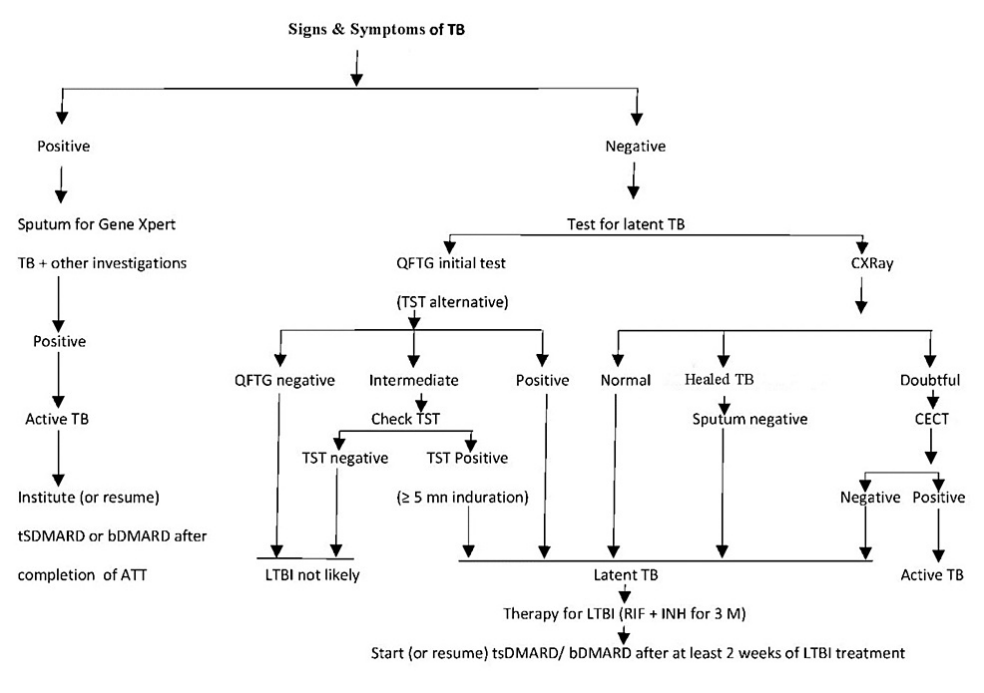
Approach for LTBI CECT, contrast-enhanced computed tomography; TST, tuberculin skin test; QFTG, QuantiFERON-TB Gold plus assay; Healed TB, only calcification or fibrotic lesions (fibrotic linear opacity, reticular fibrotic scars) in the absence of radiological evidence of active TB (upper lobe consolidation, unilateral hilar lymphadenopathy, cavitary lesions). Image credit: Muhammad Shoaib Momen Majumder.

BRS recommends a comprehensive clinical evaluation of persons for active TB (e.g., lymph nodes, spine for gibbus deformity, epididymis for epididymitis) if TST, IGRA, or CXR is positive. BRS advises three months of isoniazid (INH) and rifampicin (RIF) combination therapy for latent TB. INH or RIF monotherapy may cause drug-resistant TB in a TB-burdened country like Bangladesh [91]. The dose is INH 5 mg/kg and RIF 10 mg/kg body weight [87] or Rimstar 2-FDC (local name of a fixed drug combination). Treatment with tsDMARD or biologics can be introduced or resumed at least two weeks after treating LTBI [92,93]. Once QFTG or TST is positive at baseline, it is expected to remain positive after the successful completion of treatment, so retesting is not required [7]. Figure 2 shows the latent TB evaluation algorithm [87,89]. 

Hepatitis B is a highly infectious disease, and immunosuppressants enhance hepatitis B virus (HBV) reactivation. HBV screening requires both HBsAg and anti-HBc total. As most HBV reactivation is asymptomatic, laboratory parameters like appearance/increase in HBV-DNA or seroconversion from HBsAg negative/anti-HBc positive to HBsAg positive make the diagnosis [94]. HBsAg-negative/anti-HBc-positive patients should be initiated prophylactic antiviral treatment (tenofovir or entecavir preferred) one week before initiating bDMARDs or tsDMARDs. Prophylactic antiviral therapy should be continued throughout treatment and stopped at least six months after tsDMARD or bDMARD discontinuation (or 12 months after rituximab completion) [95].

Recommendation 11

Vaccination is recommended in all RA patients treated with DMARDs or biologics. RA is associated with an increased risk of infection and infection-associated mortality. BRS strongly recommends yearly influenza, pneumococcal, and hepatitis B vaccines (all are non-live vaccines) for all RA patients. These vaccines should be provided before the bDMARD initiation and can be given during csDMARD and tsDMARD therapy [7,96]. Vaccine administration should be completed four weeks before B cell therapy, like rituximab [96]. The schedule of pneumococcal vaccines is provided in Table 6. Two doses of recombinant HZ vaccine (RZV, Shingrix) with a shorter interval are required, regardless of previous shingles (from ≥19 years of age) [97]. Multiple vaccinations can be administered on the same day, e.g. influenza and pneumococcal vaccines with or without the hepatitis B vaccine [98]. Live vaccines in newborns should be delayed until six months of age if a pregnant woman receives biologics for severe RA in the third trimester, such as using RTX in the third trimester, etanercept beyond 32 weeks, and adalimumab beyond 28 weeks [35]. The infant should receive the BCG and OPV (oral polio vaccine) vaccination in the sixth month or later (typically given at six weeks), and the rest of the immunizations can be administered on schedule. As RA patients are immunocompromised, an additional fourth dose of the HBV vaccine should be considered. Post-vaccine antibody response testing is indicated, particularly before initiating tsDMARDs and bDMARDs. A booster HBV vaccine dose should be administered with anti-HbsAg <10 mIU/mL [99].

**Table 6:** Schedule of different vaccines for RA patients RA, rheumatoid arthritis; NA, not applicable.

Vaccine name	Dose 1	Dose 2	Dose 3	Dose 4
Influenza vaccine	Annually single dose			
Hepatitis B vaccine (Engerix-B/Hepa-B 2 mL each dose as two 1-mL doses)	1st dose	One month after dose 1	Two months after dose 1	On month 6
Pneumococcal conjugate vaccine (PCV)	Single dose in lifetime			
Pneumococcal polysaccharide vaccine (PPSV)	1st dose	Five years after dose 1	At 65 years of age	NA
Schedule of PCV13 and PPSV23 vaccines	PCV13→After 8 weeks PPSV23 (1st dose)→5 years PPSV23 (2nd dose)→At age 65 (3rd dose)	PPSV23→1 year interval PCV13→Repeat PPSV23 5 years after 1st PPSV23 →At age 65 (final dose)	NA	
Recombinant Zoster Vaccine (Shingrix)	1st dose	1-2 months after the 1st dose	NA	NA

Recommendation 12

The treatment of RA should be continued for an indefinite period. Tapering of DMARD should only be considered if the RA target remission is maintained for at least six months. Planning for tapering should only be done if the patient achieves sustained remission without GCs. Sustained remission requires six months on DAS28 <2.6 without GCs [100]. Early diagnosis and treatment, more effective DMARDs, and a treat-to-target strategy have made clinical remission a realistic goal for RA treatment. BRS advises gradual DMARD tapering to minimize flares. The tapering schedule for csDMARDs can be done for six months, with half dose in the initial three months and a quarter dose in the later three months, and then maintained or discontinued [101]. For those on MTX-SSZ combination or triple therapy, gradual withdrawal of SSZ is preferred over MTX due to higher adverse effects with SSZ [102] and cost. According to the TARA trial, tapering of bDMARDs (anti-TNF) was not superior to csDMARDs who are on csDMARD and bDMARD combination therapy; BRS suggests tapering of bDMARD first for the higher cost [101]. ACR recommends gradual tapering (or discontinuation) of MTX who achieved sustained remission with the MTX tsDMARD/bDMARD combination [103]. Those who maintain remission on a tapering dose of a single DMARD and have no relapse risk factors may discontinue DMARDs. The predictors of relapse are the female sex, longer disease duration, higher baseline DAS28 scores, and positive antibodies (rheumatoid factor or anti-CCP antibody) [104]. These patients should be monitored for flare and those who experience flare after DMARD tapering (or discontinuation) can be controlled after reintroducing the original anti-rheumatic schedule [104]. 

Discussion

Treatment of RA is a dynamic process as research continuously provides new information. BRS recommendations are based on current clinical trials and the evidence from the updated literature. The BRS developed its first management recommendations to increase physicians’ awareness and help them make the most appropriate decisions they commonly encounter during their clinical practice. This recommendation addressed several new issues unique to European and American recommendations. MTX remains the initial drug for the treatment of RA. It has discussed the dosage, administration, and management intolerance of MTX. The use of different csDMARDs, including LEF, SSZ, and HCQ (those who are MTX intolerant or have a contraindication) in special situations, including pregnancy and comorbidities, is also covered. Steroid monotherapy is strongly discouraged and emphasizes the need to protect against GC-related adverse effects, e.g., osteoporosis, Cushing syndrome, CVDs, infection, and so on. All the voting panel members were firmly in favor of this recommendation. One of the unique features of this recommendation is to apply a tsDMARD for patients who had a failure or intolerance with MTX or a csDMARD. The tsDMARDs were preferred to bDMARDs for lower cost and easier availability in Bangladesh. Subsequently, we considered the possibility of transitioning to an alternative tsDMARD or a biologic agent in the event of initial tsDMARD failure. Another unique recommendation was incorporating the Padua Prediction Score to assess the risk for thromboembolic risk prediction for those requiring dose escalation of the tsDMARDs, as the less thrombogenic bDMARDs are far more expensive. We have discussed the dosage and selection of different tsDMARDs in different comorbidities and the adverse events with their risk factors.

As infection is more prevalent in Bangladesh, including TB, clinical and laboratory vigilance carries high importance when using DMARDs, particularly tsDMARDs. We have mentioned that an algorithm for screening for latent TB has been recommended strongly before starting the tsDMARD and bDMARDs and discussed the preferred latent TB therapy for the Bangladeshi population. IGRA was preferred; however, TST or Mantoux test has been recommended instead of IGRA if the test is unavailable or cost becomes a concern in those situations. The voting panel favored the combination of RIF and INH for three months, considering the high risk of resistance due to anti-TB monotherapy in Bangladesh. The TF and SC discussed the indefinite continuation of DMARDs and cautious discontinuation of drugs, mainly those who achieved sustained remission for at least six months. Most of the recommendations are entrenched in a high level of evidence and strength, except the recommendations for vaccination part.

The vaccination statement was uncategorized and remained ungraded due to the inclusion of different vaccination options in a single recommendation. Although a general agreement was quickly established on most recommendations, two specific difficulties necessitated extended deliberation and argumentation. A comprehensive discussion took place to determine an appropriate method for assessing the thromboembolic risk in individuals using tsDMARDs. Ultimately, the voting panel reached a consensus on the Padua Prediction Score due to its simplicity and reliance on readily available clinical information. The TST cutoff value at 5 mm was a topic of dispute. Ultimately, the TF and SC have reached a consensus on utilizing a TST threshold of greater than 5 mm to diagnose LTBI in individuals with RA, taking into account the level of immune suppression and recent studies completed in East Asian countries. The significant impact of RA on health outcomes and economic factors underscores the necessity of ensuring public health and prioritizing this condition within the framework of national health policy. 

## Conclusions

The BRS management recommendation may be important in providing evidence-based, updated treatment recommendations for RA management in a resource-constrained setting. BRS plans to review and update these recommendations in the future.

## Appendices

**Table 7:** Categories of evidence [5]

Category	Evidence
1A	From a meta-analysis of randomized controlled trials
1B	From at least one randomized controlled trial
2A	From at least one controlled study without randomization
2B	From at least one type of quasi-experimental study
3	From descriptive studies, such as comparative studies, correlation studies, or case-control studies
4	From expert committee reports or opinions and/or clinical experience of respected authorities

**Table 8:** Strength of recommendations [5]

Strength	Directly based on
A	Category 1 evidence
B	Category 2 evidence or extrapolated recommendations from category 1 evidence
C	Category 3 evidence or extrapolated recommendation from category 1 or 2 evidence
D	Category 4 evidence or extrapolated recommendation from category 2 or 3 evidence

**Table 9:** 2010 ACR/EULAR classification criteria for the diagnosis of RA [6] NA, not applicable; RF, rheumatoid factor; ACPA, anti-citrullinated peptide antibody; ESR, erythrocyte sedimentation rate; CRP, C-reactive protein. The maximum scores from each of the domains A through D are added, and the sum is considered the total score. ^a^Total score of ≥6 is needed to classify a patient as having definite RA. A joint involvement refers to any swollen or tender joint on examination, which may be confirmed by imaging evidence of synovitis. DIP joints, first CMC joints, and first MTP joints are excluded from the assessment. Large joints refer to shoulders, elbows, hips, knees, and ankles. Small joints refer to MCP joints, PIP joints, second through fifth MTP joints, thumb IP joints, and wrists. ^b^Negative means less than or equal to the upper limit of normal (ULN); low positive means >ULN but <3 ULN; high positive means >3 ULN. ^c^Normal and abnormal are determined by local laboratory standards. ^d^Duration of symptoms taken as per the patient’s self-report.

Domain	Category	Point Score
A	Joint involvement (05 points)^a^	NA
	1 large joint	0
	2-10 large joints	1
	1-3 small joints (large joints not counted)	2
	4-10 small joints (large joints not counted)	3
	>10 joints, including at least one small joint	4
B	Serology (at least one test needed for classification; 03 points)^b^	NA
	Negative RF and negative ACPA	0
	Low-positive RF or low-positive ACPA	2
	High-positive RF or high-positive ACPA	3
C	Acute-phase reactants (at least one test needed for classification; 0-1 point)^c^	NA
	Normal CRP and normal ESR	0
	Abnormal CRP or abnormal ESR	1
D	Duration of symptoms	NA
	<6 weeks	0
	>6 weeks	1
